# Biomaterial-based therapeutic strategies for oral submucous fibrosis

**DOI:** 10.3389/fcell.2026.1891450

**Published:** 2026-08-24

**Authors:** Yue Su, Yinyi Song, Fan Xue, Hanbing Wang, Zitong Zeng, Yourong Jiang

**Affiliations:** Department of Stomatology, Jining Medical University, Jining, China

**Keywords:** biomaterial, cell therapy, drug delivery system, exosome, oral submucous fibrosis

## Abstract

Oral submucous fibrosis (OSF) is a chronic, potentially malignant oral mucosal disease driven by areca nut chewing. It is characterized by progressive collagen deposition and debilitating symptoms such as trismus and burning sensation, which severely impair quality of life and confer a risk of malignant transformation. Given the limited efficacy of current therapies, this review aims to provide a comprehensive and critical appraisal of the evolving therapeutic landscape for OSF. We summarize conventional strategies, including pharmacological, surgical and physical approaches, alongside emerging therapeutic strategies, through a comprehensive literature review and screening of historical periodicals. The analysis reveals that established treatments have inherent limitations, whereas emerging approaches, including biomaterial-based delivery systems, nucleic acid and cell-based therapies, drug-loaded microneedle patches, and preventive interventions, show promise by overcoming poor mucosal retention, limited epithelial penetration and rapid salivary clearance. Mechanistic insights and the therapeutic potential of these strategies are discussed based on preclinical evidence. We conclude that current OSF therapies have limited efficacy, underscoring the need for innovative approaches. Emerging biomaterial-based systems and targeted drug delivery hold substantial potential, but further clinical research is essential to validate their safety and effectiveness for patient care.

## 1 Introduction

Oral submucous fibrosis (OSF) is a chronic, progressive, potentially malignant disorder characterized by pathological collagen deposition in the submucosal connective tissue, leading to epithelial atrophy, mucosal rigidity, and progressive trismus (Wei et al., 2024; Kondaiah et al., 2019).

Clinically, patients suffer from burning sensation, xerostomia, and dysphagia, which severely compromise quality of life (Fang et al., 2019; Mokal et al., 2025). With malignant transformation rates reportedly ranging from 15% to 30%, OSF represents a significant oncological concern (Zhang et al., 2022). The fibrotic microenvironment, characterized by tissue hypoxia, chronic inflammation, and extracellular matrix stiffening, actively contributes to carcinogenesis through multiple mechanisms including epithelial-mesenchymal transition, activation of mechanotransduction pathways such as yes-associated protein (YAP)/transcriptional coactivator with PDZ-binding motif (TAZ), and dysregulation of transforming growth factor-β (TGF-β) signaling (Raha et al., 2026; Vasundra et al., 2026). Notably, the stiffened matrix in OSF has been shown to promote a migratory phenotype and drive fibrosis-associated type 2 epithelial-mesenchymal transition (EMT) toward type 3 EMT, which is closely linked to malignancy (Shetty et al., 2025). The etiology is multifactorial, with areca nut chewing identified as the primary risk factor, alongside genetic predisposition, nutritional deficiencies, and immune dysregulation (Chiewwit et al., 2024; Liu et al., 2022; Wu et al., 2023; Bijai and Muthukrishnan, 2022; Ranganathan and Kavitha, 2025; Yuwanati et al., 2023).

Despite advances in understanding OSF pathogenesis, effective disease-modifying therapies remain scarce. Histopathologically, OSF is defined by dysregulated collagen metabolism, fibroblast hyperactivation, and impaired vascularization (Zhi et al., 2024; Yang et al., 2024). Current clinical management conventionally relies on pharmacological interventions, physical therapy, and surgical release of fibrotic bands (Xu et al., 2025; Rao et al., 2020; Shih et al., 2019; Chitlange and Phansopkar, 2023). However, these approaches are largely palliative: drug therapy is hindered by poor mucosal retention, inadequate epithelial penetration, and rapid clearance by salivary flow; physical therapy offers only symptomatic relief; and surgical intervention is invasive, often associated with recurrence and postoperative scarring. Critically, none of these modalities reverse the underlying fibrotic process or restore normal tissue architecture.

These therapeutic constraints have galvanized intensive investigation into material-based strategies engineered to overcome the formidable barriers of the oral mucosa. The stratified squamous epithelium, intercellular tight junctions, and incessant salivary flow present obstacles that conventional formulations fail to surmount. Recent innovations in biomaterials and nanomedicine have yielded sophisticated platforms expressly designed for oral mucosal drug delivery, encompassing hydrogels with programmable retention kinetics, mucoadhesive microneedle patches achieving minimally invasive barrier penetration, and nanoformulations that augment drug stability and mucosal permeability. Parallel advancements in deciphering OSF pathogenesis have enabled the emergence of nucleic acid therapeutics capable of precisely modulating pro-fibrotic signaling networks, alongside stem cell-derived exosome therapies that harness paracrine signals to orchestrate resolution of the fibrotic niche.

While existing reviews have addressed OSF pathogenesis and conventional treatments, a comparative synthesis of biomaterial-based therapeutic platforms for OSF is currently lacking. Against this background, the present review provides a critical appraisal of these emerging therapeutic paradigms, examining their capacity for sustained and spatially controlled drug release, enhanced mucosal permeability, and cell-free modulation of fibrotic processes. By integrating contemporary preclinical and translational evidence, this review aims to provide a unified conceptual framework for understanding the potential of material-driven interventions in OSF, whilst identifying pivotal challenges and opportunities that will shape future therapeutic strategies for this disease.

## 2 Methodology

**FIGURE 1:**
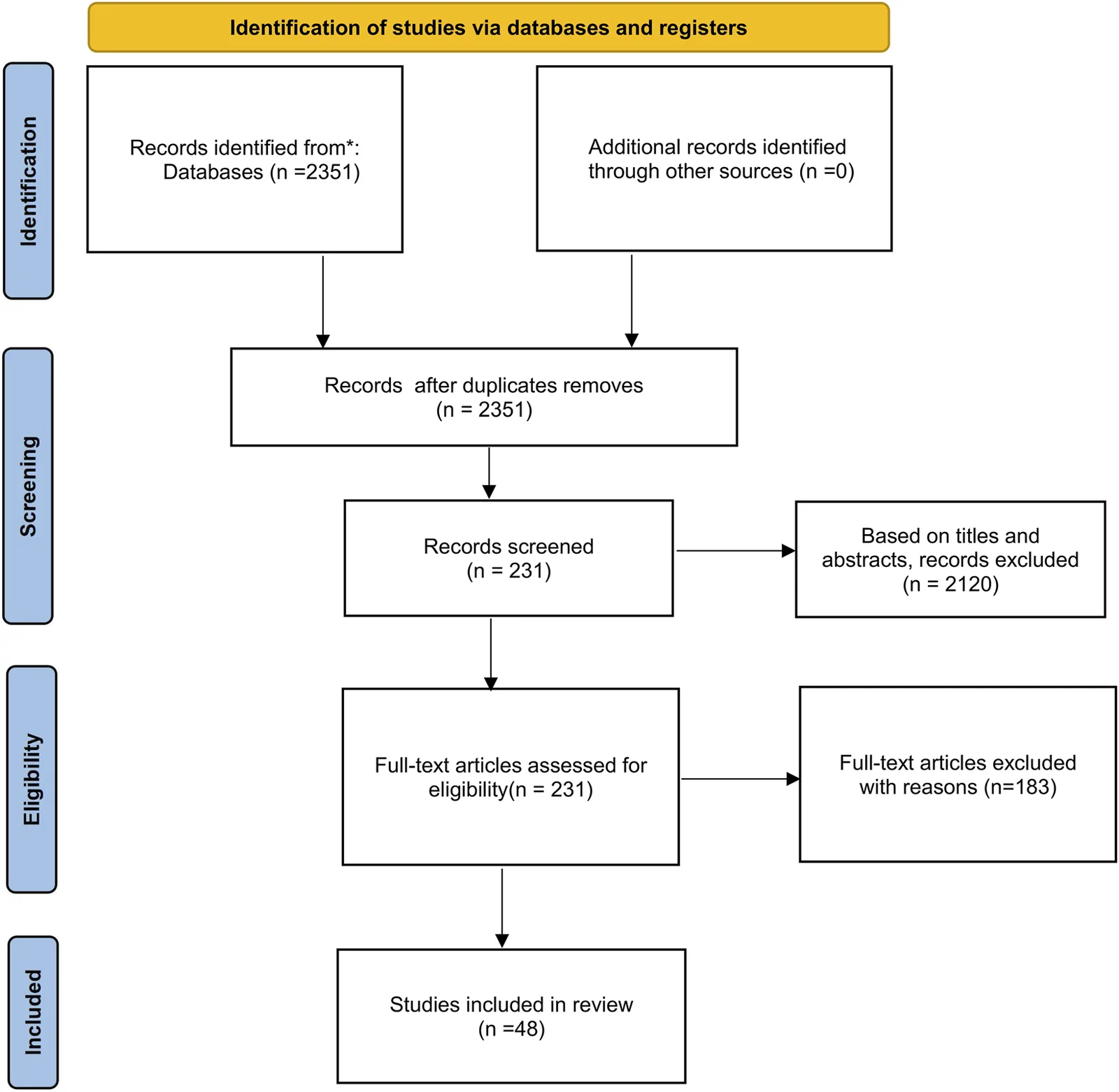
Flow chart of the selection process.

We primarily searched the PubMed database using Boolean operators (AND, OR) to combine keywords, including “oral submucous fibrosis”, “OSF”, “biomaterial”, “drug delivery system”, “nanoparticle”, “hydrogel”, “microneedle”, “nucleic acid therapy”, “cell therapy”, “exosome”, “mesenchymal stem cell” and related terms. The search timeframe was restricted from January 2019 to May 2026. Inclusion criteria were set to original research, reviews and clinical studies that focused on therapeutic strategies for OSF; exclusion criteria comprised non-English publications, conference abstracts, case reports with insufficient data, and studies unrelated to OSF treatment. The initial search yielded 2,351 articles. After preliminary screening by reading titles and abstracts, articles that did not meet the thematic criteria or were duplicates were removed, resulting in a pool of 231 articles. These were further assessed for eligibility through full-text reading, and ultimately 48 articles were selected and included in the qualitative synthesis. The complete process of literature retrieval and study selection is documented in detail in the PRISMA flow diagram, as shown in Figure 1.

## 3 Biomaterial-based therapeutic strategies

The profound limitations of conventional management have galvanized the emergence of sophisticated biomaterial-based platforms, including hydrogels, microneedle patches, nanoformulations, nucleic acid therapeutics, and exosome-based interventions, that transcend conventional palliation by enabling sustained drug retention, epithelial barrier penetration, and targeted modulation of fibrotic pathways.

### 3.1 Biomaterial delivery systems

**FIGURE 2:**
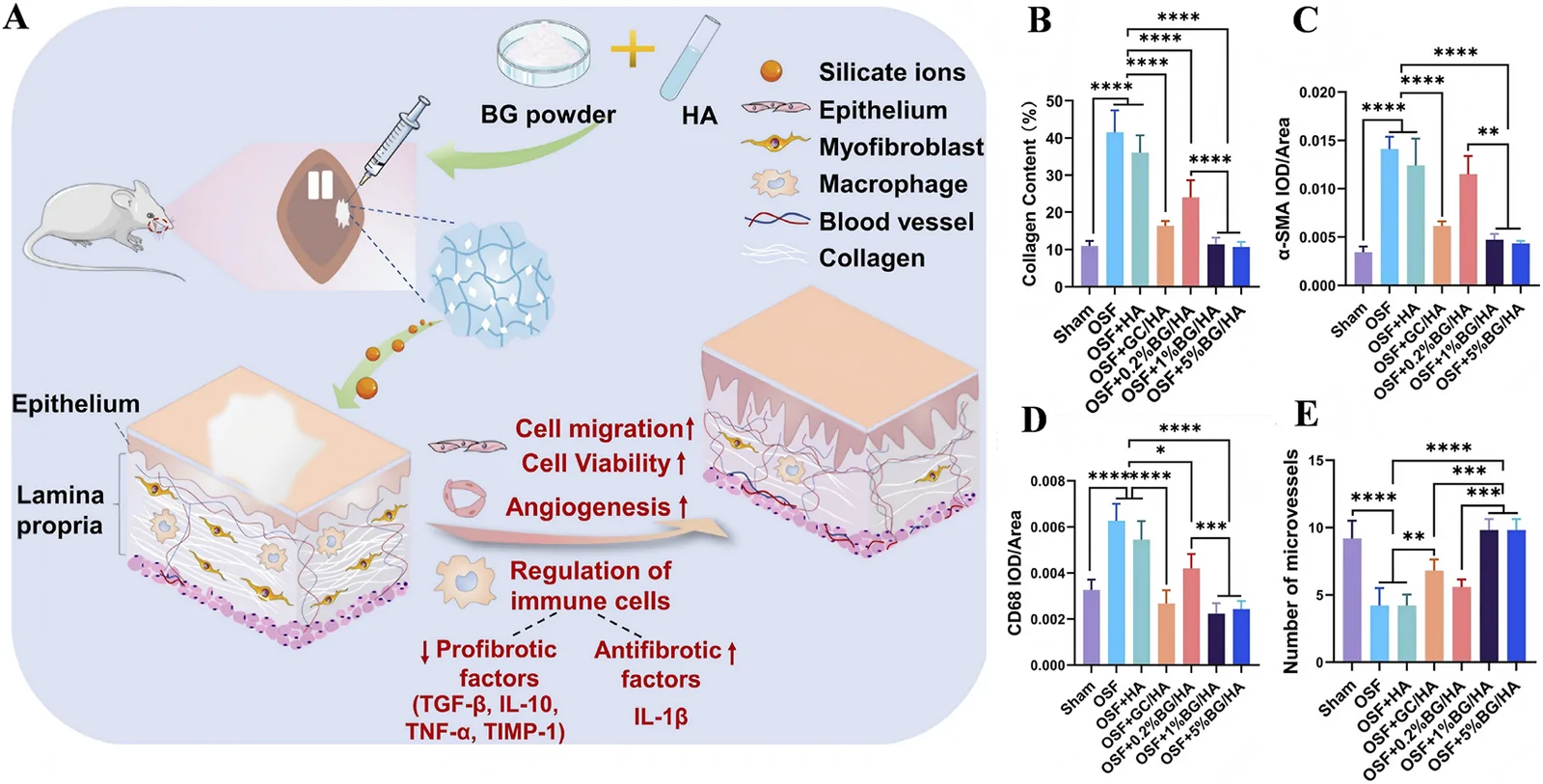
Therapeutic effects of BG/HA composite hydrogel on betel-alkaloid-induced rat OSF. **(A)** Mechanistic hypothesis of BG/HA treatment for OSF. **(B)** Quantitative collagen content based on Masson’s trichrome staining. Representative immunohistochemical images and quantitative analysis of α-SMA **(C)**, CD68 **(D)**, and CD31 **(E)** in oral mucosal tissues from different groups *in vivo* (n = 6) (Guo et al., 2023). Copyright 2023, Elsevier.

Nanomaterials demonstrate notable properties, including a high surface area-to-volume ratio, increased electrical conductivity, superparamagnetism, spectral shifts in light absorption, and unique fluorescence features, and are typically within the size range of 1–100 nm (Cheng et al., 2021). As drug carriers, they offer several advantages, such as improved drug stability, controlled drug release, high tissue permeability, and precise targeting capabilities (Birk et al., 2021; Chen and Hu, 2024). These nanomaterial carriers effectively encapsulate drugs, minimizing the risk of sudden release, extending retention times, and facilitating rapid penetration through the oral mucosa. In one investigation, a nano-hydrogel engineered for delivering epigallocatechin gallate (EGCG) to treat OSF was evaluated. Its positively charged surface facilitates interaction with the negatively charged oral mucosa, leading to high mucosal permeability. In addition, it improved oral mucosal conditions and mouth opening by reducing TGF-β1 and collagen levels, without exhibiting toxicity to major organs or mucosal tissues (Mehta et al., 2024). Hydrogels, given their favorable biocompatibility, biodegradability, drug-loading capacity, and sustained drug release properties (Yang et al., 2022), are a viable alternative for drug delivery systems. As an illustration, the researchers developed an injectable composite hydrogel based on sodium hyaluronate and 45S5 bioglass (BG/HA) (Figure 2A). When administered into fibrotic regions, it markedly suppresses excessive collagen deposition via sustained release of bioactive silicate ions. The hydrogel effectively attenuates inflammation driven by extensive macrophage infiltration by directly modulating macrophage functional states. It downregulates the secretion of pro-fibrotic mediators, including TGF-β1, IL-10, TNF-α, and tissue inhibitor of metalloproteinase (TIMP)-1, while upregulating anti-fibrotic cytokines such as IL-1β. Simultaneously, this ionic solution enhances epithelial cell viability and migration capacity, stimulates vascular endothelial cells to promote capillary angiogenesis and mucosal epithelial repair, thereby effectively delaying fibrosis progression (Figures 2B–E) (Guo et al., 2023).

With innovations in dosage forms, research has advanced from matrix systems reliant on passive diffusion to microneedle patches capable of actively penetrating physiological barriers. Researchers have developed more efficient, minimally invasive, and painless microneedle patches for oral mucosal drug delivery to overcome the limitations of traditional oral mucosal drug delivery methods—such as the short duration of drug presence on the mucosal surface, difficulties in penetrating the epithelial barrier, and discomfort associated with injections. Recent studies have concentrated on creating drug-loaded microneedle patches for treating OSF, utilizing triamcinolone acetonide integrated with a fibroin/tannic acid mixture and a fibroin suspension. These patches enable sustained drug release, maintaining efficacy for up to 7 days. Compared with conventional commercial oral patches, innovative drug-loaded microneedle patches offer significantly improved wet adhesion strength, demonstrate no cytotoxicity, and facilitate effective drug penetration into the lamina propria of the mucosa. Consequently, they ensure that the therapeutic agent is delivered directly to the lesion site in OSF, thereby potentially enhancing treatment outcomes (Cheng et al., 2023). Furthermore, the microneedle patch with pre-assembled sericin protein nanospheres (SFN) facilitates sustained and precise release of triamcinolone at the lesion site through multiple mechanisms. These include electrostatic and hydrophobic interactions between SFN and triamcinolone, the physical barrier formed by the nanospheres, and dual diffusion pathways of the drug within both the SFN and sericin protein matrix. The SFN significantly extends the drug’s active duration from 4 days to 14 days, covering typical clinical injection intervals while enabling painless delivery across the epithelial barrier to the core fibrotic area. Consequently, this system provides an innovative solution for long-acting treatment, improving patient compliance, and enhancing the efficacy of OSF therapy (Cheng et al., 2024).

Additionally, spray-form nanomedicines demonstrate significant potential in enhancing drug retention and penetration, offering another effective approach for the local treatment of OSF. The epigallocatechin gallate (EGCG)-loaded NanoCuboSpray (NCS) promotes sustained drug release by improving mucosal adhesion and penetration via positively charged nanocubes. EGCG NCS significantly improves oral opening range and epithelial thickness while effectively downregulating TGF-β1 and type I collagen expression, thereby promoting symptomatic improvement in OSF (Mehta et al., 2025).

Despite these promising preclinical findings, translating biomaterial platforms into clinical practice for OSF requires overcoming several critical hurdles. Issues such as scalable manufacturing under GMP conditions, long-term biocompatibility assessment, and sterilization methods compatible with hydrogel integrity have yet to be systematically investigated. In addition, the regulatory pathway for microneedle patches remains ambiguous, particularly given their classification as combination products that may require evaluation by multiple regulatory bodies.

In addition to the aforementioned platforms, antioxidant and catalytic nanomaterial platforms have emerged as a class of nanomaterials with potential relevance to OSF. These materials, represented by SOD/CAT-mimetic nanozymes based on Ce, Mn, Co, Pt, Fe, and Cu, effectively scavenge ROS and restore redox homeostasis (Luo et al., 2025). Among these, CeO_2_ nanoparticles represent one of the earliest and most extensively characterized platforms in this context. Owing to the reversible Ce^3+^/Ce^4+^ valence transition on their surface, nanoceria exhibit potent and self-regenerating ROS scavenging activity (Matussin et al., 2023). Notably, these platforms have demonstrated anti-fibrotic efficacy across multiple fibrotic disease models. In liver fibrosis, CeO_2_ nanoparticles have been shown to downregulate TGF-β1, phosphorylated mothers against decapentaplegic homolog (Smad)2/3, collagen (COL)1A1, matrix metalloproteinases (MMP)2, and α-SMA expression in hepatic tissues (Mowaad et al., 2026). In myocardial infarction models, CeO_2_/Nrf2 nanocomposites alleviated cardiac systolic dysfunction and significantly reduced infarct size and scar formation (Liao et al., 2024). In intestinal fibrosis, an oral engineered microsphere system encapsulating hollow mesoporous CeO_2_ nanoparticles achieved ROS scavenging efficiency exceeding 95% and reduced α-SMA expression by 66% (Qiao et al., 2025). Given the well established role of oxidative stress in OSF pathogenesis, the integration of such catalytic nanomaterials into mucoadhesive delivery systems for OSF represents a promising yet unexplored therapeutic avenue, although direct evidence in OSF models remains to be established and warrants further investigation.

### 3.2 Nucleic acid therapeutics

Nucleic acid-based therapeutics represent a promising strategy for precision medicine by directly modulating the expression of disease-associated genes. The successful implementation of this approach, however, is contingent upon overcoming a central challenge, namely, the safe and efficient delivery of inherently unstable therapeutic nucleic acids such as microRNA (miRNA) mimics or inhibitors to specific target cells. To address this limitation, nanomaterial-based carriers, including liposomes and polymers, have been widely adopted to encapsulate and transport anti-fibrotic miRNAs, thereby enhancing their molecular stability and ensuring effective delivery to effector cells (Manikkath et al., 2022).

**FIGURE 3:**
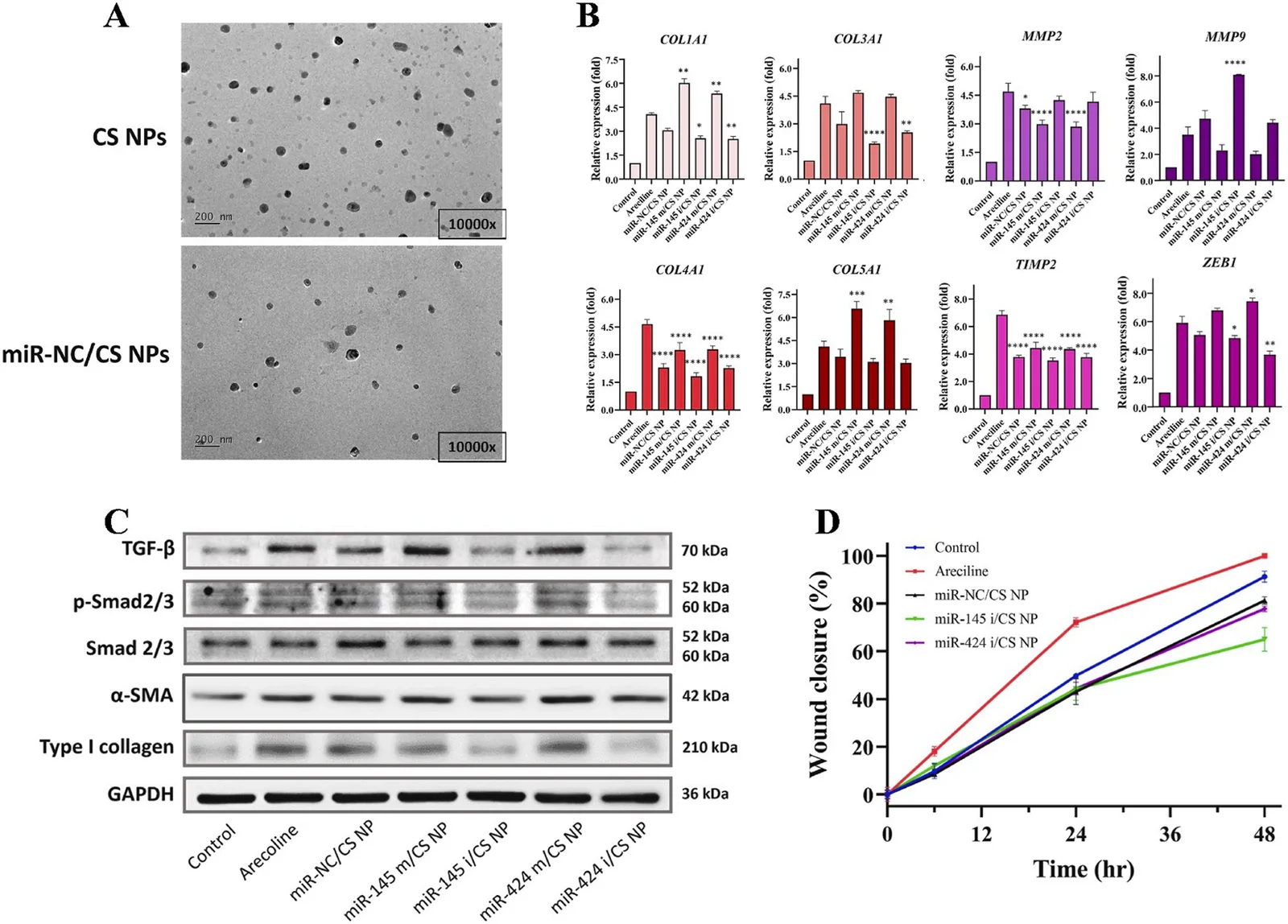
Nucleic acid therapeutics for OSF. **(A)** TEM inspection to the blank CS NPs and miR-NC/CS. **(B)** mRNA expression profile of arecoline-stimulated oral submucosal fibroblasts transfected with miR-NC, miR-145, and miR-424 by CS NPs. **(C)** Western blot analysis of the transfected fibroblasts. **(D)** Wound healing assay of transfected normal and arecoline-stimulated oral submucosal fibroblasts (Cheng et al., 2025). Copyright 2025, Elsevier.

Substantial progress has been made in recent years in developing sophisticated delivery platforms based on such nanocarriers. For instance, chitosan nanoparticles (CS NPs) have been validated as efficient vehicles for delivering inhibitors of miR-145 and miR-424 into arecoline-stimulated oral submucosal fibroblasts. This co-delivery strategy synergistically blocks the TGF-β signaling pathway, resulting in the downregulation of key fibrotic markers (TGFB1, ACTA2, COL1A1, COL3A1, COL4A1, MMP2, TIMP2, ZEB1), inhibition of cell migration, and consequent suppression of wound closure ability, which highlights its potential as a therapeutic intervention for OSF (Figure 3) (Cheng et al., 2025).

To enhance therapeutic benefits additionally, the strategy of co-transfection of multiple anti-fibrotic miRNAs has been demonstrated to exert a synergistic potentiation effect. Recent studies have substantiated that the simultaneous delivery of miR-29a-3p, miR-196a-3p, and miR-509-5p mimics potently suppresses the expression of key collagen genes, including COL1A1, COL3A1, and COL5A1, concurrent with the downregulation of pivotal matrix remodeling regulators such as MMP1, MMP7, TIMP1, and TIMP2, thereby efficiently inhibiting the production of phosphorylated Smad (pSmad)2/3, α-smooth muscle actin (α-SMA), and type I collagen protein. Notably, this combinatorial regimen exerts a markedly more profound inhibitory effect on the TGF-β/Smads signaling pathway than single-miRNA transfection. Consequently, it demonstrates superior efficacy in diminishing the production of extracellular matrix components, inhibiting wound closure, and suppressing collagen gel contraction (Fang et al., 2025).

Although nucleic acid therapeutics enable precise modulation of fibrotic pathways, several unresolved challenges continue to impede their clinical advancement. These include inefficient *in vivo* delivery, potential immunogenicity, and the complete absence of safety data in human oral mucosa. Until these issues are adequately addressed, clinical evaluation will remain premature.

### 3.3 Cell therapies

**FIGURE 4:**
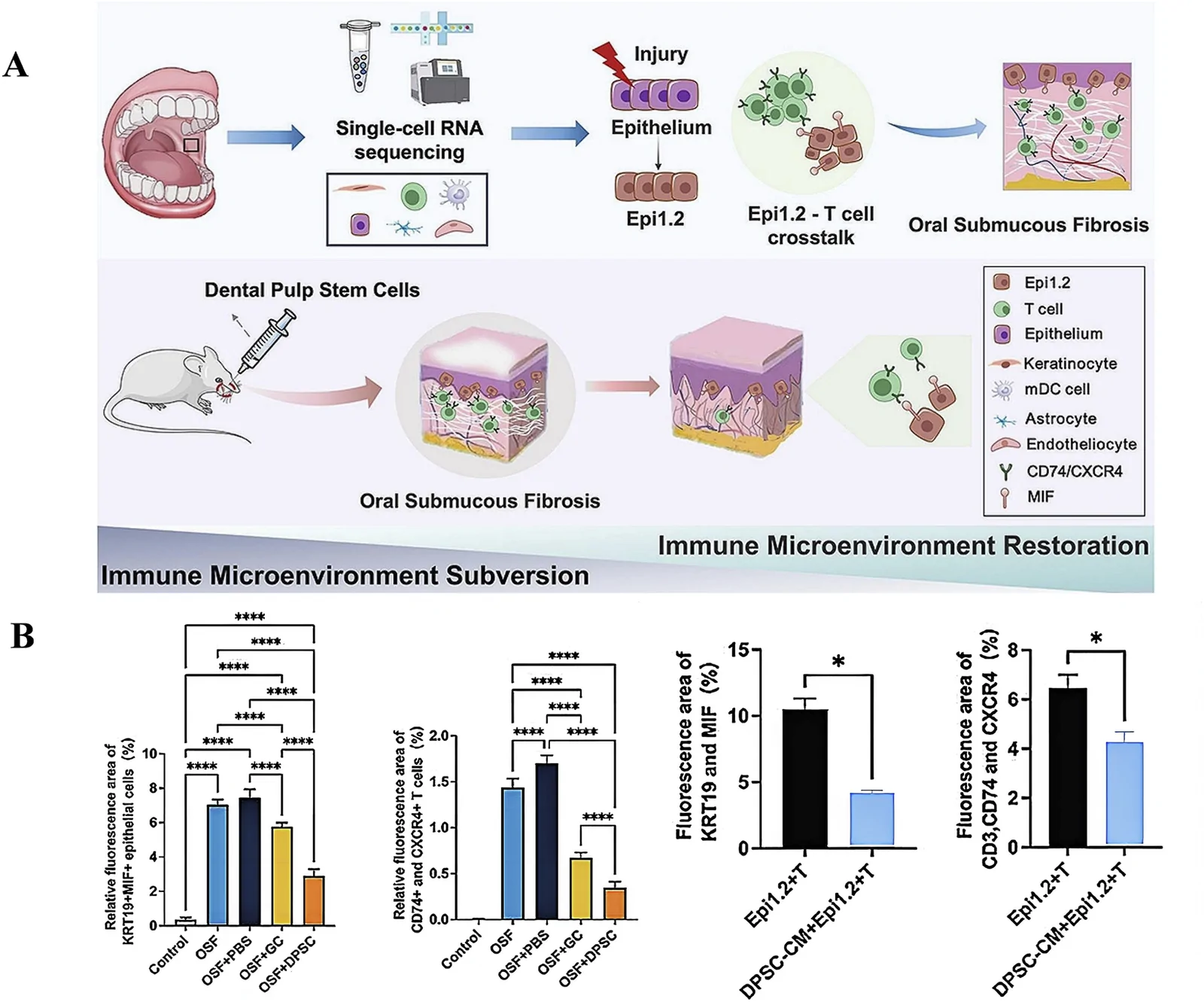
Mechanism of DPSC-based therapy disrupting pathological epithelial–T cell crosstalk in OSF. **(A)** Schematic illustration of the mechanism of action for DPSC-based local delivery therapy. **(B)** DPSCs block aberrant epithelial–T cell communication by inhibiting the Epi1.2–MIF–CD74/CXCR4 signaling axis (Wang et al., 2024). Copyright 2024, Springer Nature.

Cell therapy represents a promising therapeutic strategy for cancers, autoimmune diseases, neurodegenerative disorders, and cardiovascular conditions, owing to its precise targeting capacity, favorable biosafety profile, sustained endogenous drug release, advanced sensing functions, and efficient immunomodulatory activities (Bashor et al., 2022). In the management of OSF, dental pulp-derived mesenchymal stem cells (DPSCs) and their secretome have been identified as possessing considerable therapeutic potential. Following local injection into the oral mucosa of OSF rat models, DPSCs were shown to effectively downregulate a pro-inflammatory and pro-fibrotic epithelial cell subset (Epi1.2) and its associated ligand MIF, disrupt pathological crosstalk with T cells, promote the reestablishment of mucosal homeostasis, and remodel the local immune microenvironment, thereby attenuating OSF progression (Figure 4) (Wang et al., 2024). Upon delivery to fibrotic tissues, DPSCs secrete bioactive factors, including hepatocyte growth factor (HGF) and interleukin-10 (IL-10), through paracrine signaling, thereby suppressing fibroblast hyperactivation and aberrant collagen deposition, promoting angiogenesis, and ultimately restoring mucosal tissue homeostasis in oral submucous fibrosis (Narvekar et al., 2025).

To improve delivery efficiency and therapeutic durability, a chitosan-based hydrogel encapsulating the DPSCs has been developed. This hydrogel is enriched with pro-angiogenic growth factors, and preclinical studies in animal models have demonstrated that its application restores normal oral mucosal epithelium, substantially reduces the expression of fibrosis-related genes (COL1, COL3, α-SMA) and oxidative stress markers (LDH, MDA, SOD), concurrently significantly enhancing mouth opening. This results in notable reversal of fibrotic phenotype and supports tissue regeneration (Mante et al., 2025).

The regenerative capacity of cell therapy is undoubtedly attractive. Nevertheless, substantial obstacles remain on the path to clinical adoption, including prohibitive production costs, rigorous GMP compliance demands, and a lack of standardized protocols for cell expansion and quality characterization. These manufacturing and regulatory barriers have yet to be resolved in a manner suitable for routine clinical application.

### 3.4 Stem cell exosome therapies

Exosomes, a pivotal class of extracellular vesicles secreted by eukaryotic cells, play an indispensable role in mediating intercellular communication and signal transduction (Dai et al., 2020). Accumulating studies have highlighted the therapeutic potential of adipose-derived mesenchymal stem cell-derived exosomes (ADSC-Exos) in OSF. These vesicles have been demonstrated to target critical pro-fibrotic signaling pathways, such as TGF-β, effectively reversing the aberrant activation of myofibroblasts and suppressing excessive collagen deposition (Renu, 2025; Wang et al., 2023).

**FIGURE 5:**
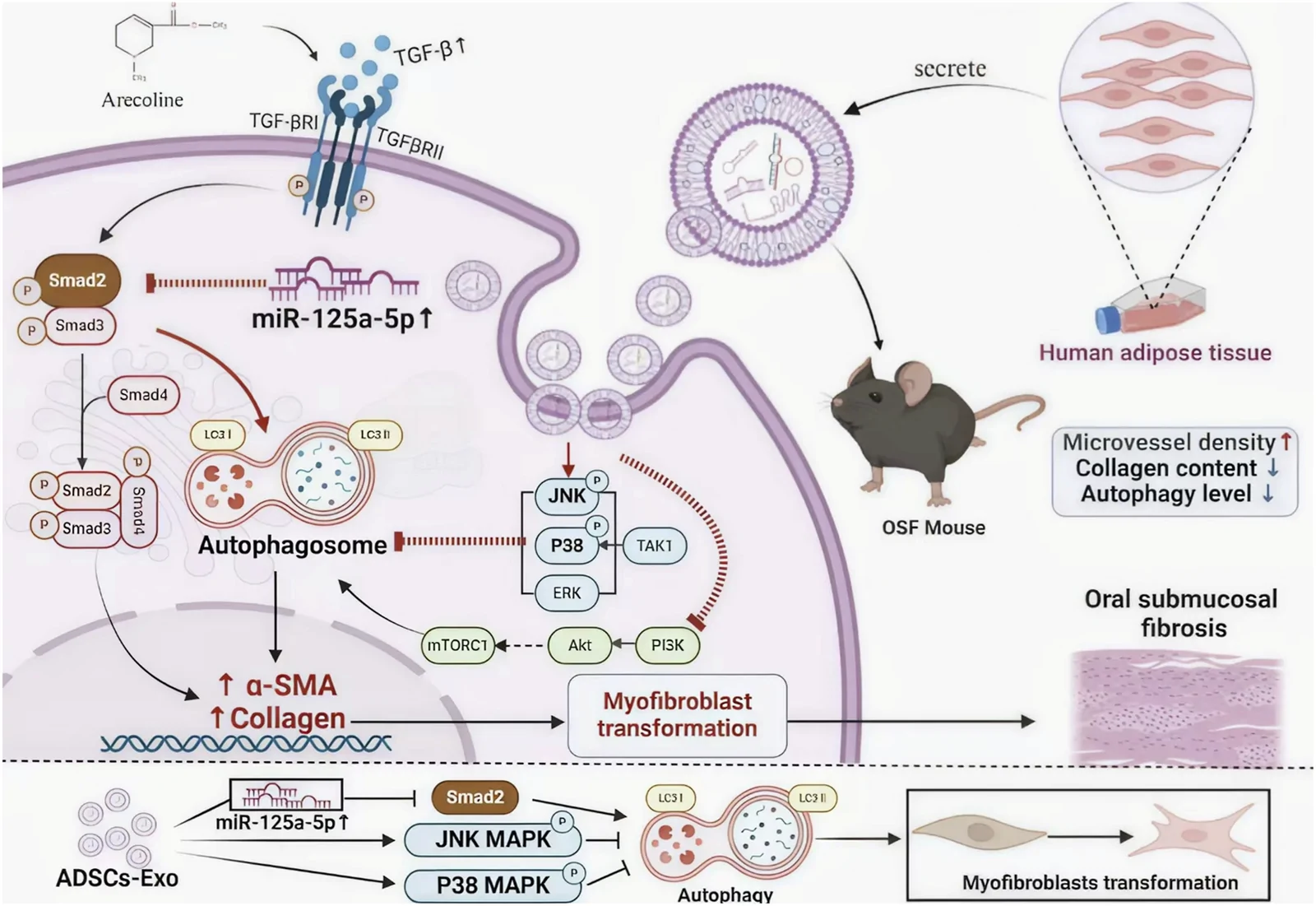
The potential mechanism of ADSC-Exo regulating myofibroblast transformation in OSF (Xu et al., 2024). Copyright 2024, Springer Nature.

The antifibrotic actions of ADSC-Exos are largely mediated by their diverse miRNA cargo, which orchestrates the regulation of the TGF-β signaling pathway and its downstream fibrogenic effects through distinct molecular axes. A prime example is miR-181a-5p, which is enriched in ADSC-Exos and can be efficiently internalized by oral mucosal myofibroblasts. By directly targeting Smad2, miR-181a-5p inhibits the activation of the TGF-β pathway, attenuates Smad2/3 phosphorylation, and impedes the subsequent formation of the Smad2/3-Smad4 transcriptional complex and its nuclear translocation. Consequently, this leads to the alleviation of collagen overaccumulation, the downregulation of key fibrotic markers including α-smooth muscle actin (α-SMA) and collagens I and III, and the reversal of myofibroblast dysregulation (Shao et al., 2024; Renu, 2025). Further supporting this paradigm, research by Han et al. revealed that exosomes overexpressing miR-375, upon internalization by buccal mucosal fibroblasts, significantly suppressed cell proliferation, migration, and invasion while promoting apoptosis. Concomitantly, a reduction in the expression levels of α-SMA and collagens I/III was observed, effectively ameliorating OSF-associated pathological phenotypes (Han et al., 2021). The pathological activation of myofibroblasts in OSF is frequently accompanied by elevated autophagy. miR-125a-5p, another miRNA constituent of ADSC-Exos, directly targets and inhibits Smad2. This interaction blocks both the TGF-β/Smad2 signaling axis and the autophagy activation mediated by it, thereby suppressing myofibroblast transdifferentiation and collagen synthesis (Xu et al., 2024). Consistent with these *in vitro* findings, local administration of ADSC-Exos in OSF model mice was shown to markedly reduce submucosal collagen fiber deposition, alongside a downregulation of key autophagy-related proteins (Figure 5) (Xu et al., 2024).

Moreover, a distinct mechanism involves the delivery of miR-760-3p by ADSC-Exos to fibrotic buccal mucosal fibroblasts. This miRNA directly targets and represses the expression of insulin-like growth factor 1 receptor (IGF1R), thereby obstructing the activation of the TGF-β1/Smad3 pathway. Both *in vitro* and *in vivo* experiments have corroborated that this axis significantly inhibits the activation, migration, and invasion of myofibroblasts, reduces the expression of α-SMA and collagens I/III, and ultimately retards the pathological progression of OSF (Wang et al., 2023).

Exosome-based therapies offer distinct cell-free advantages over living cell products. However, their clinical translation is constrained by considerable challenges in large-scale production, isolation purity, batch-to-batch consistency, and long-term stability under typical storage conditions. The establishment of standardized characterization and potency assays represents a prerequisite for meaningful clinical development.

The comparative features of these platforms are summarized in Table 1.

**TABLE 1:** Comparative summary of biomaterial-based therapeutic platforms for OSF.

Platform	Characteristics	Key findings	Advantages	Limitations
Hydrogel systems	Bioglass-containing hyaluronate hydrogel for sustained local ion release	↓ Collagen deposition, ↓ inflammation (TGF-β1, IL-10, TNF-α), ↑ angiogenesis and epithelial repair (Guo et al., 2023)	Biocompatible, injectable, sustained release	GMP scale-up and sterilization not addressed
Microneedle patches	Mucoadhesive patch with drug-loaded microneedles for epithelial penetration	Sustained release up to 7 days (Cheng et al., 2023) and up to 14 days (Cheng et al., 2024), painless delivery to lamina propria	Minimally invasive, prolonged efficacy, high compliance	Mechanical strength, regulatory classification unclear
Nanoformulations	Positively charged nanocubes with enhanced mucosal adhesion and penetration	↑ Mouth opening, ↑ epithelial thickness, ↓ TGF-β1 and COL1A1 (Mehta et al., 2025)	Enhanced mucosal retention and permeability	Long-term safety, manufacturing complexity
Nucleic acid therapeutics	Chitosan nanoparticle-mediated delivery of miRNA mimics/inhibitors to fibroblasts	↓ Fibrotic markers (TGFB1, COL1A1, ACTA2, MMP2), ↓ migration, synergistic effects (Cheng et al., 2025)	Precision targeting, synergistic multi-miRNA effects	Delivery efficiency, immunogenicity, stability
Cell therapy	Local injection of dental pulp-derived MSCs; immunomodulation via paracrine signaling	↓ Epi1.2-MIF axis, disruption of epithelial-T cell crosstalk, tissue regeneration (Wang et al., 2024)	Regenerative potential, multi-target modulation	High cost, GMP compliance, batch variability
Exosome therapy	Extracellular vesicles delivering anti-fibrotic miRNAs (e.g., miR-181a-5p) to myofibroblasts	↓ α-SMA, ↓ collagens I/III via TGF-β/Smad2 axis (Shao et al., 2024); ↓ autophagy (Xu et al., 2024)	Cell-free, low immunogenicity, stable miRNA cargo	Scalability, purity, storage stability

**FIGURE 6:**
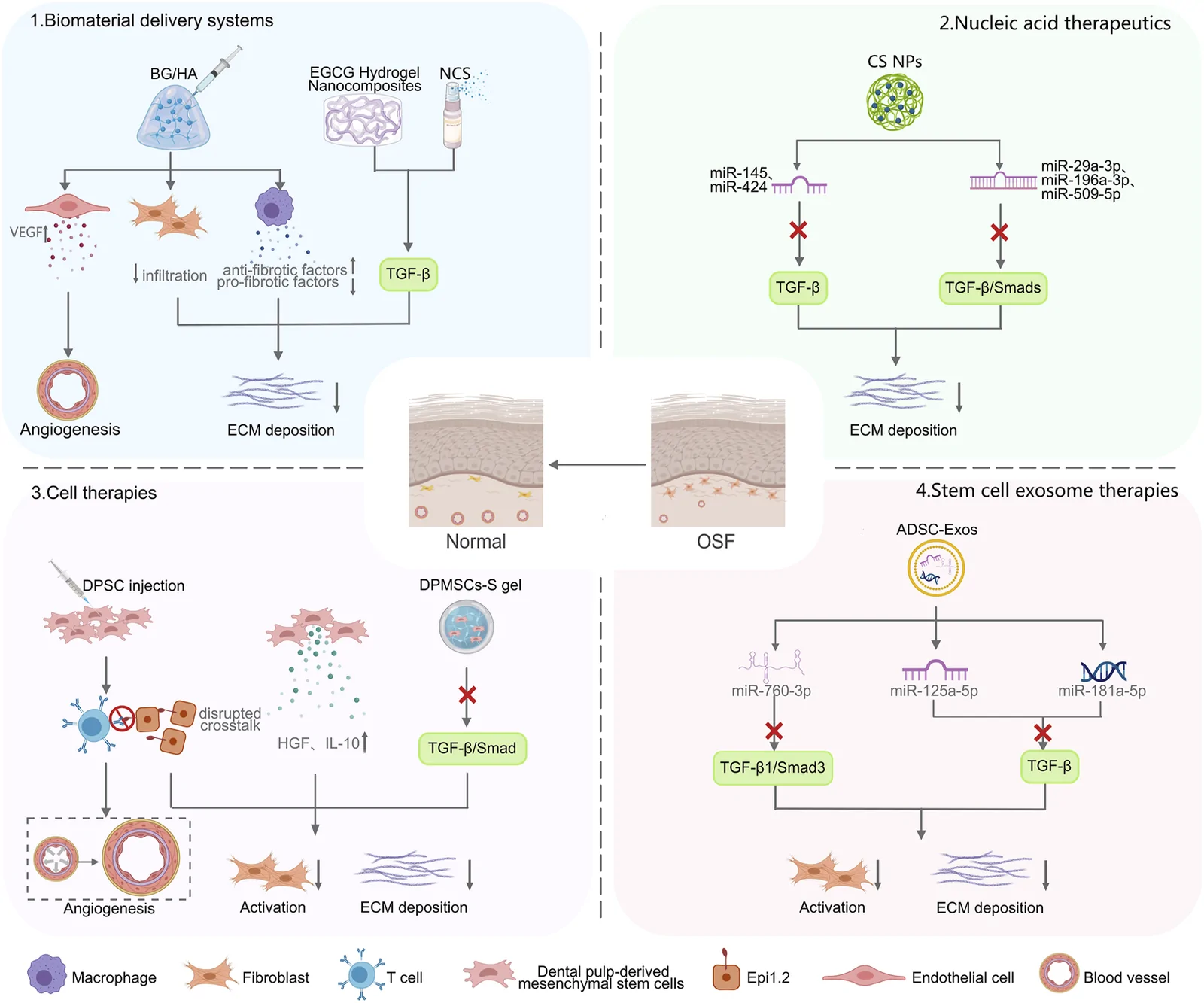
Schematic diagram of biomaterial-based therapeutic strategies for OSF. (1) Biomaterial delivery systems: BG/HA composite hydrogel modulates immune cell function, EGCG nanocomposites and NCS reduce extracellular matrix deposition and promote angiogenesis by inhibiting TGF-β-mediated fibrogenesis. (2) Nucleic acid therapeutics employ chitosan nanoparticles as carriers to deliver antifibrotic miRNAs, blocking the TGF-β/Smads pathway and suppressing excessive collagen production. (3) Dental pulp-derived mesenchymal stem cell-based therapies disrupt pathological epithelial–T cell crosstalk and inhibit fibroblast hyperactivation via paracrine bioactive factors. (4) Stem cell exosome therapies deliver functional miRNAs targeting key nodes of the TGF-β signaling axis to reverse myofibroblast activation and halt fibrotic progression.

Based on the above discussion, the therapeutic landscape and its integration with OSF pathogenesis are illustrated in Figure 6.

## 4 Conclusion and future perspectives

OSF remains a challenging condition with limited curative options. Conventional therapies, including corticosteroids, physical therapy, and surgery, are largely palliative, constrained by poor mucosal retention, inadequate epithelial penetration, and inability to reverse established fibrosis. Emerging biomaterial-based strategies offer promising alternatives. Hydrogels and scaffolds enable sustained localized drug release, while microneedle patches facilitate minimally invasive epithelial penetration and nanoformulations enhance drug stability and mucosal permeability. Nucleic acid therapeutics delivered via nanocarriers and stem cell-derived exosome therapies further expand the therapeutic arsenal, with preclinical evidence demonstrating reduced collagen deposition and attenuated myofibroblast activation. Despite these advances, no biomaterial-based system has yet entered clinical trials for OSF; manufacturing scalability, regulatory classification, and cost-effectiveness therefore await rigorous evaluation. Future research should prioritize elucidating OSF pathogenesis, optimizing material properties, developing clinically relevant animal models, and bridging preclinical innovation to clinical application. Given the recognized contribution of fibrotic pathology to carcinogenesis, the potential relationship between sustained resolution of OSF pathology and reduced long-term malignant transformation rates also warrants investigation. Addressing these challenges will enable mechanism-driven, material-enabled interventions that not only alleviate symptoms but ultimately reverse fibrosis and improve patient outcomes.

Several interconnected directions merit attention. Smart biomaterials capable of responding to pathological cues, such as elevated reactive oxygen species, acidic pH, or protease activity, as well as external stimuli including near-infrared light and temperature, could enable on-demand drug release specifically within fibrotic tissues. For instance, hydrogels incorporating oxidation-labile linkers, such as thioketal or phenylboronic acid moieties, may facilitate selective release of antifibrotic agents in regions of sustained oxidative stress. Concurrent advances in artificial intelligence can expedite formulation design by predicting polymer-drug compatibility, optimizing nanoparticle surface properties for mucosal permeation, and extracting structure-activity relationships from existing data, thereby reducing dependence on empirical screening.

The transition toward clinical application will likely be shaped by personalized and combinatorial approaches. Patient-derived organotypic models and disease specific biomarkers may inform platform selection according to individual disease severity and molecular profiles. Engineered extracellular vesicles enriched with antifibrotic microRNAs, such as miR-181a-5p or miR-760-3p, or loaded with small molecule inhibitors, offer targeted modulation while circumventing the regulatory complexities associated with live cell products. Multimodal regimens co-delivering nucleic acid therapeutics and small-molecule drugs within a single carrier, or integrating biomaterial-based delivery with photobiomodulation, could address the multifactorial pathogenesis of OSF more effectively than any monotherapy.

Ultimately, successful translation will depend on several interdependent factors. These include animal models that faithfully recapitulate the progressive fibrosis, epithelial atrophy, and immune dysregulation characteristic of human OSF. Early regulatory consultation to delineate approval pathways for combination products is also essential, as are rigorous safety, biocompatibility, and cost-effectiveness assessments relative to existing corticosteroid regimens. Sustained interdisciplinary collaboration among materials scientists, clinicians, pharmacologists, and regulatory experts will be equally critical. Achievement of these objectives would enable mechanism-driven, material-based interventions capable of not merely palliating symptoms but fundamentally reversing fibrotic progression and improving long term patient outcomes.

## Glossary

OSF: Oral submucous fibrosis
YAP: Yes-associated protein
TAZ: Transcriptional coactivator with PDZ-binding motif
TGF-β: Transforming growth factor beta
EMT: Epithelial-mesenchymal transition
EGCG: Epigallocatechin gallate
BG/HA: Sodium hyaluronate and 45S5 bioglass
NCS: NanoCuboSpray
ROS: Reactive oxygen species
COL: Collagen
α-SMA: α-smooth muscle actin
miRNAs: MicroRNAs
CS NPs: Chitosan nanoparticles
MMP: Matrix metalloproteinase
TIMP: Tissue inhibitors of matrix metalloproteinase
DPSCs: Dental pulp-derived mesenchymal stem cells
IL: Interleukin
ADSC-Exos: Adipose-derived mesenchymal stem cell-derived exosomes
Smad: Mothers against decapentaplegic homolog
IGF1R: Insulin-like growth factor 1 receptor
TEM: Transmission electron microscopy
ECM: Extracellular matrix
SFN: Sericin protein nanospheres
p-Smad: Phosphorylated Smad
ZEB1: Zinc finger E-box binding homeobox 1
Exos: Exosomes
